# A Simple Non-Embedded Single Capillary Device for On-Demand Complex Emulsion Formation

**DOI:** 10.3390/mi15020239

**Published:** 2024-02-04

**Authors:** Mohammad Mahdi Karim Khani, Mehrnaz Oveysi, Vahid Bazargan, Marco Marengo

**Affiliations:** 1 School of Mechanical Engineering, College of Engineering, University of Tehran, Tehran 4399-57131, Iran; mmahdi.kkhani@ut.ac.ir (M.M.K.K.); mehrnaz.oveysi@ut.ac.ir (M.O.); 2 Department of Civil Engineering and Architecture, University of Pavia, 27100 Pavia, Italy

**Keywords:** double emulsion, complex emulsion, capillary

## Abstract

This study includes an examination of the design, fabrication, and experimentation of a rudimentary droplet generator. The device has potential applications in on-demand double and higher-order emulsions as well as tailored emulsions with numerous cores. The phenomenon of a pendant double droplet creation is observed when an inner phase is transported through a capillary, while a middle phase envelops the external surface of the capillary. This leads to the occurrence of a pinching-off process at the tip of the pulled capillary. Following this, the double droplet is introduced into a container that is filled with the outer phase. The present study examines the force equilibrium throughout the droplet break-up process and aims to forecast the final morphology of the droplets within the container by considering the impact of interfacial tension ratios. The shell thickness in a core–shell formation can be calculated based on the inner and middle phase flow rates as well as the middle droplet formation period. The present platform, which enables the simple production of double and higher emulsions, exhibits promising prospects for the controlled manufacturing of complex emulsions. This technology holds potential for various applications, including the experimental exploration of collision behavior or electro-hydrodynamics in emulsions as well as millimeter-size engineered microparticle fabrication.

## 1. Introduction

There exist multiple techniques for the generation of double emulsions, which include two-step emulsification utilizing mechanical stirring, a vibrating mixer, or a homogenizer [1]. The field of microfluidics has revolutionized the control and precision in the fabrication of emulsions, as highlighted in Wang’s work on microfluidic systems [2]. Employing planar devices like flow-focusing setups and three-dimensional capillary-based structures has become a common practice for generating controlled higher-order and complex emulsions, as evidenced by the research of Choi, Leister, and Tenorio [3,4,5]. However, it is crucial to recognize that these configurations, especially when utilized for producing complex and multiple emulsions, come with certain limitations and challenges. These include the need for specific surface treatments, the necessity for specialized additives or surfactants tailored to each fluid, the intricate fabrication process often requiring clean-room facilities, and the meticulous alignment required for capillary-based devices [6,7]. Furthermore, incorporating flow channels at the micro-scale level for emulsion production with a length scale of less than 500 μm not only escalates costs but also introduces challenges related to device clogging [8]. Despite their widespread applications [9,10,11,12], emulsions exceeding the 500-micrometer length scale have received relatively limited attention. This points to an intriguing area with potential for further exploration and innovation in the field of millifluidics.

Confined flows were used as millifluidic droplet generators of complex emulsions production in several studies [13,14,15,16]. To effectively generate larger droplets in co-flow devices, it is imperative to decrease the flow rate of the outer phase. The phenomenon of droplet accumulation in stationary locations is frequently observed as a result of the reduction in flow rate, mostly attributed to the gravitational force acting on horizontal devices [17]. Scholars have endeavored to address this constraint using several methodologies, with a specific focus on investigating vertically aligned co-axial capillary configurations, as expounded upon in the study conducted by [17,18,19,20]. In the vertical configurations, the uniformity of droplet size was compromised due to the uncontrolled rupture of a compound jet. Another constraint of this approach is the restriction of thin-shell emulsion fabrication [17]. Exploration into vertically oriented co-axial capillary arrangements offers a promising avenue for addressing these challenges and advancing the production of complex structures.

In this study, we introduce a novel single-vertical capillary device that boasts both ease of fabrication and simplicity, yet possesses the remarkable capability for gravity-based fabrication of complex and high-order emulsions with high precision. What sets this device apart is its unique feature—the emulsions it generates do not require the addition of surfactants or alteration of the device’s surface to manipulate wettability. This basic apparatus represents a significant advancement in millimeter-sized emulsion production, offering a straightforward yet highly effective approach for achieving controlled and monodispersed emulsions. This apparatus is especially advantageous when it comes to generating tailored complex or higher-order emulsions, especially in scenarios such as the experimental exploration of electro hydrodynamics in emulsions, as discussed in [21], or the experimental examination of the collision behavior of intricate emulsions under different environmental conditions, as explored in [22].

This study offers a comprehensive examination, both theoretically and experimentally, of the controlled generation of several different double emulsions using the drop maker apparatus. The device is utilized to generate triple emulsion drops and multiple core emulsion drops to demonstrate its additional potential. Furthermore, the technique employed for the accumulation of dual droplets in the external phase is utilized in the production of hollow alginate microparticles, which are recognized as an exceptional carrier for encapsulating water-soluble substances [23]. This method is significantly simpler compared to previous studies that have documented the generation of 3D hollow structures using various techniques, including electrospinning [24], elimination of the core through calcination [25], and emulsification [18].

## 2. Materials and Methods

### 2.1. Emulsion Preparation

The materials used in this study were sourced as follows. Properties are assigned to materials based on the information provided by their labels. Sesame oil (with a density of 919 kg/m^3^ and a viscosity of 34 cSt) was procured from a local store, while silicone oil (with a density of 950 kg/m^3^ and a viscosity of 20 cSt) was acquired from Sigma-Aldrich. HFE 7500 (with a density of 1614 kg/m^3^ and a viscosity of 0.77 cSt) was purchased from 3M. De-ionized water, characterized by a density of 998 kg/m^3^ and a viscosity of 1 cSt, was obtained from a purification system.

The Jikan CAG-20 (Jikan Surface Nano-Engineering Company, Tehran, Iran) an automated apparatus for quantifying static and dynamic contact angles, as well as surface and interfacial tensions, was employed to determine the interfacial tension between the two phases at ambient temperature. The measurement of interfacial tension between water and sesame oil yields a result of 23.7 mN/m. The interfacial tension between HFE-7500 (3M, Maplewood, MN, USA) and water is measured to be 32.2 mN/m, while the interfacial tension between silicone oil and water is found to be 40.5 mN/m. For hollow microparticle preparation, sodium alginate (Product number W201502) and PLGA 75:25 (poly(lactide-co-glycolide), $M_{w}$ 66,000–107,000) were procured from Sigma-Aldrich (St. Louis, MO, USA) to create hollow microparticles. The rotating rheometer (AR 1500ex, TA Instruments, New Castle, DE, USA) is used to quantify the viscosity of the alginate solution. The kinematic viscosity of sodium alginate in water at a temperature of 25 °C was measured to be 19.4 cSt. The level of inaccuracy was found to be approximately ±0.05 cSt. The organic solvent, namely dichloromethane (DCM), and acetic acid were purchased from Merck Millipore (Darmstadt, Germany). The calcium carbonate nanopowder was acquired from FineNano (Mashhad, Iran) To improve visualization, Oil Red O, a fat-soluble dye purchased from Sigma, was dissolved in the oil phase. Furthermore, green or red food dyes, sourced from a local retailer at a concentration of 0.05 wt%, were dissolved in the aqueous phase.

### 2.2. Device

**Figure 1:**
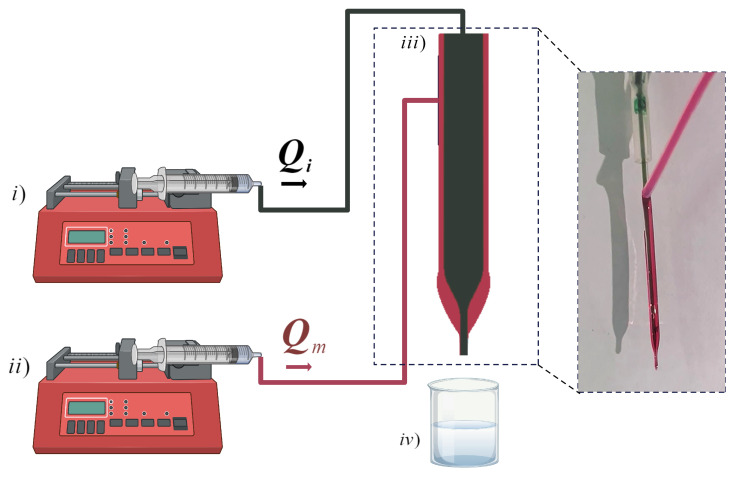
Schematic of designed vertical capillary device for droplet formation. (i.) Inner phase, (ii.) Middle phase injection for wetting the outer surface of the capillary. (iii.) In a capillary, the inner phase is flowing inside it and the outer phase is wetting the outside of it. (iv.) A container filled with the outer phase. In addition to the schematic, the figure also incorporates an actual image of the device.

The double droplet generator setup features a glass capillary with a circular design, possessing an internal diameter of 0.3 mm and an external diameter of 1.32 mm. The capillary is elongated to create a tip with an external diameter of 0.2 mm. The inner phase was introduced into the system via the inner capillary at a volumetric flow rate $Q_{i}$. The external surface of the capillary is only wetted by the middle phase at a volumetric flow rate $Q_{m}$. The break-up of the pendant double droplet happens at the tip of the capillary, as shown in Figure 1. To generate a triple droplet, a dental needle, specifically a 30-gauge needle with an inner diameter of 0.140 mm, was inserted into a glass capillary until it stopped just short of reaching the capillary’s end. It was intentionally positioned slightly higher to facilitate the passage of flows. The outer fluid was then injected to cover the outer surface of the capillary. The separation of the triple droplet occurred at the tip of the capillary. To examine the process of droplet production, a camera was employed to capture the several stages of double droplet development and subsequent pinch-off. The droplet that had undergone pinch-off was introduced into a container containing 5 mL of the outer phase. The drop size measurements were conducted in the downstream region, where the drops achieved their final morphology. To determine the average size of each emulsion, a minimum of 10 drops were measured. Frames were analyzed using the ImageJ software (1.8.0, LOCI, University of Wisconsin).

To generate a triple droplet, a dental needle, specifically a 30-gauge needle with an inner diameter of 0.14 mm, was inserted into the glass capillary until it reached the capillary’s end. This created a simultaneous flow of two fluids—one inside the needle and the other between the needle and the capillary. Subsequently, the external fluid was introduced to envelop the external surface of the capillary. The pendant triple droplet separated at the capillary tip, and the ultimate morphology was achieved within the container containing the outer phase.

#### Emulsion and Microparticle Preparation

The introduction of fluids into the microfluidic apparatus was executed through PTFE tubing. This process was facilitated by a syringe pump, specifically the Harvard PHD model, which ensured precise control over the flow rate as per the experimental requirements. The fluids utilized in this study were stored in 5 mL syringes (Luer slip syringe needle, AVA Pezeshk, Tehran, Iran). To investigate the behavior of fluid–fluid interactions within the apparatus, emulsions of water-in-oil-in-oil (w/o/o), oil-in-water-in-oil (o/w/o), and water-in-oil-in-water (w/o/w) were generated. The triple emulsion of water-in-oil-in-oil-in-water (w/o/o/w) can be fabricated with minor adjustments to the equipment. Hollow polymeric microparticles are generated through the utilization of a 1 wt% PLGA in dichloromethane as the inner phase and 1 wt% aqueous sodium alginate solution as the middle phase. The pendant double droplets are pinched off from the capillary tip. The container, into which droplets are dripped, entails a dispersion of 0.5 M Calcium carbonate nanopowder in HFE-7500. Following the process of double droplet collection, the outer phase is supplemented with 0.5 mL acetic acid to initiate the gelation of alginate. The present approach for internal gelation is based on the methodology outlined in the work conducted by [26], with several modifications.

## 3. Droplet Formation Mechanism

**Figure 2:**
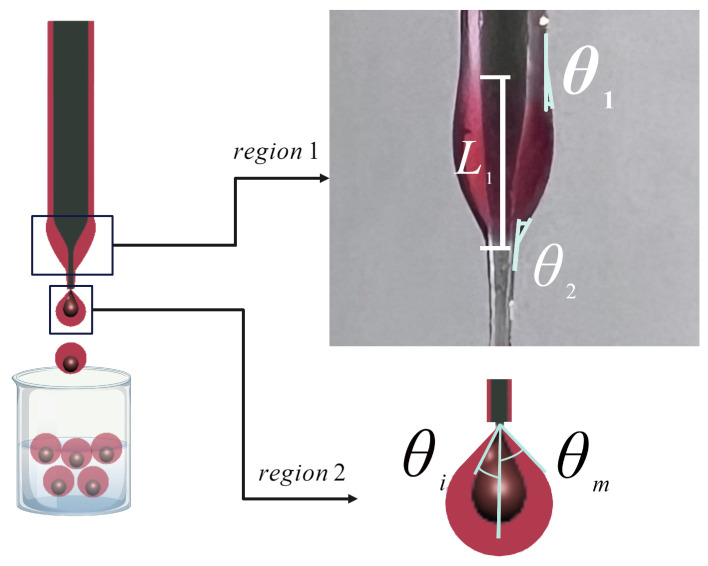
Double droplet of water in oil evolution with time.

**Figure 3:**
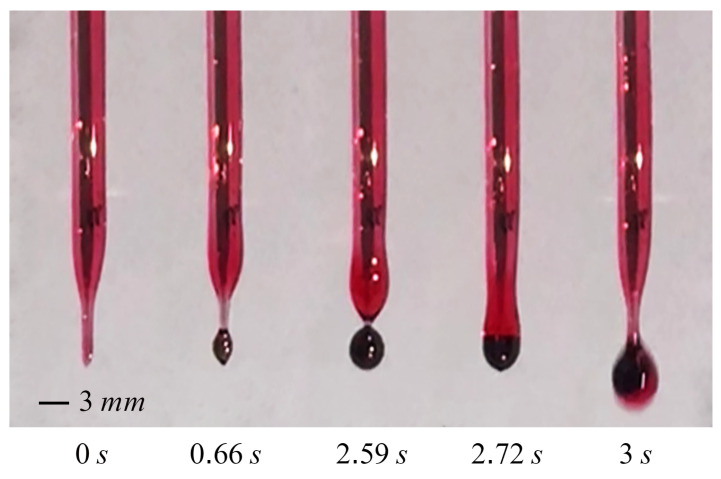
According to the second case (Case 2), the formation of a double droplet (water in oil) over time is observed.

The device is specifically engineered to generate emulsions of double and higher orders. The process of emulsion creation begins with the detachment of pendent double or triple droplets at the tip of the capillary. These droplets then fall into a container that is filled with the outer phase and the ultimate morphology of the emulsion is attained. The pinch-off mechanism is governed by the flow of the middle phase along the external surface of the capillary. Hence, the wetting characteristics of the capillary tube, the interfacial tension between the phases, the viscosity of the phases, and the surface tension of the middle phase are all parameters that influence the pinch-off behavior. The geometry of the capillary (after the pulling) influences the motion of the middle phase along the external surface of the capillary. In our experiments, we observed two distinct pinching-off models. The flow behavior of the middle and inner phases exhibits dissimilar patterns in two particular regions (region 1 and 2), as depicted in Figure 2. In the first case, the inner phase gathers at the end of the capillary, while the outside surface of the capillary is completely wet by the middle phase, resulting in the accumulation of the middle phase at the tip as well. Pinch-off occurs in Region 2 when both phases collect at the tip, as a result of the combined action of volume and body forces. In the alternative case (Case 2), the complete coverage of the outer surface of the capillary by the middle phase is prevented by the physical features of the middle phase, such as viscosity and surface tension. Consequently, the accumulation of the middle phase occurs in Region 1, which is different from the prior case when the accumulation took place in Region 2. Additionally, although the inner phase initially accumulates in Region 2, it subsequently rises upwards to Region 1. When the two phases come into contact, the inner phase is drawn toward the middle phase as a result of interfacial tension. Then, both fluids flow downwards toward the tip of the capillary and undergo pinch-off at Region 2. The visual representations of the second case are presented in Figure 3. To comprehensively investigate the process of droplet formation in this scenario, a recorded video is employed to evaluate the formation of double droplets, namely water droplets in sesame oil. The video is transformed into a series of images. The time evolution of the double droplets formed by the single-capillary device is illustrated in Figure 3. At a time of 0.66 s, the middle phase is absent at the apex of the capillary. This phenomenon can be ascribed to the upward motion of the middle phase that occurs after each droplet separates. Between 0.66 s and 2.59 s, the middle phase is collected in Region 1 while the inner phase travels upwards toward this region. An attracting motion of the two phases toward each other is observed at Region 1 at 2.72 s, followed by the pinching off of the capillary tip at Region 2 at 3 s.

## 4. Theoretical Methodology of Droplet Pinch-Off

**Figure 4:**
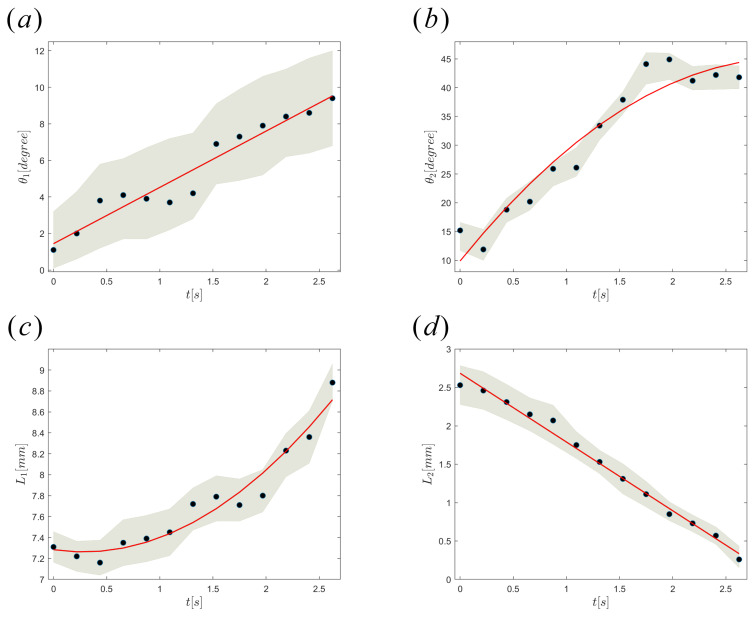
Variation of (**a**) $\theta_{1}\left(t\right)$, (**b**) $\theta_{2}\left(t\right)$, (**c**) $L_{1}\left(t\right)$, and (**d**) $L_{2}\left(t\right)$ with time. The markers represents the average value obtained from eight repeated measurements for each frame. A shaded region is employed to highlight the variability or confidence interval associated with the data.

In the context of a numerical study, the configuration of the non-embedded single-capillary system is analyzed within an axisymmetric coordinate system. Figure 2 presents a schematic illustration of the unconfined apparatus. The variable *R* represents the outside diameter of the capillary, whereas the variable *r* denotes the diameter of the capillary tip. The wetting thickness, denoted by δ, refers to the thickness of the middle phase at the location $z=h_{1}$. Here, $h_{1}$ represents the point at which capillary deformation begins due to the pulling, and it also marks the starting point of Region 1. The wetting thickness, denoted as $\delta^{*}$, corresponds to the value of *z* which represents the lower limit of region 1, where $h_{2}$ is defined. The variable $L_{1}\left(t\right)$ denotes the disparity in elevation between $h_{2}$ and $h_{1}$, and it has the potential to undergo temporal fluctuations throughout the droplet formation process. Depending on the wettability characteristic of the inner phase, the inner phase may move upward on the outer surface of the capillary tip. $h_{3}$ is showing the vertical location of the inner phase on the capillary. $L_{2}\left(t\right)$ is the vertical length defined as $L_{2}=h_{3}-h_{2}$. The contact angles $\theta_{1}\left(t\right)$ and $\theta_{2}\left(t\right)$ are the angles formed by the interface at the top and lower boundaries of Region 1 concerning the z-axis, respectively. $R_{i}$ and $R_{m}$ are the inner and outer diameters of the double pendant droplet during pinch-off. The estimation of the time-dependent parameters, namely $\theta_{1}\left(t\right)$, $\theta_{2}\left(t\right)$, $L_{1}\left(t\right)$, and $L_{2}\left(t\right)$, is based on the analysis of the temporal evolution of the interface shape. This analysis is performed using a succession of recorded images, as illustrated in Figure 4a–d. The function $\theta_{1}\left(t\right)$ exhibits a positive correlation with time as a result of the buildup of the middle phase, while $\theta_{2}\left(t\right)$ displays an increasing pattern until it reaches a steady state. This tendency aligns with the temporal variance in $L_{1}\left(t\right)$. When the function $L_{1}\left(t\right)$ experiences a variation, and the middle phase initiates flow, the variable $\theta_{2}\left(t\right)$ will cease to undergo any more changes. The observed decrease in the value of $L_{2}\left(t\right)$ can be attributed to both the upward movement of the inner phase and the downward movement of the middle phase.

### 4.1. Force Balance

During the double droplet pinch-off, the inner and middle phases are subjected to several forces. Here, we will apply our force balance models to Regions 1 and 2, contributing to cases 2 and 1, respectively.

#### 4.1.1. Force Balance on the Middle Phase at Region 1: Case 2 of Pinching-Off Mechanism

The force–balance equation is written for Region 1 as shown in (Equation (1)). Kinetic force (Equation (2)), gravity (Equation (3)) [17], and the surface tension force (Equation (4)) pull the droplet down. The opposite counteracting force is surface tension (Equation (5)). To maintain equilibrium according to the force balance equation, the variable δf is introduced and computed as indicated in Equation (6) by defining $\bar{u}\left(t\right)=\frac{Q_{m}}{\pi {\left(R+\delta \right)}^{2}-R^{2}}$. It is suggested that δf(*t*) accounts for the combined influence of the tube’s geometric characteristics and the chemical properties of the middle phase.

(1) $$F_{\sigma_{1}}=F_{g}+F_{k}+F_{\sigma_{2}}+\delta f\left(t\right)$$

(2) $$F_{g}=M\left(t\right)g=\dot{m}gt=\rho Q_{m}gt$$

(3) $$F_{k}=\rho Q_{m}\bar{u}\left(t\right)$$

(4) $$F_{\sigma_{1}}=2\pi \left(R+\delta \left(t\right)\right)\sigma_{m}cos\theta_{1}\left(t\right)$$

(5) $$F_{\sigma_{2}}=2\pi \left(r+\delta^{*}\right)\sigma_{m}cos\theta_{2}\left(t\right)$$

(6) $$\begin{array}{c} \delta f\left(t\right)=2\pi \sigma_{m}\left(\left(R+\delta \right)cos\theta_{1}\left(t\right)\right)-\left(r+\delta^{*}\right)cos\theta_{2}\left(t\right))-\\ \rho Q_{m}\left(gt+\frac{Q_{m}}{\pi {\left(R+\delta \right)}^{2}-R^{2}}\right) \end{array}$$

The magnitudes of the forces can be categorized as follows: surface tension with a magnitude of *O(1E-4)*, gravity with a magnitude of *O(1E-5)*, δf with a magnitude of *O(1E-4)* and kinetic forces with a magnitude of *O(1E-10)*. Hence, in comparison to other forces, the kinetic force can be disregarded for Region 1.

#### 4.1.2. Force Balance at Region 2: Case 1 of Pinching-Off Mechanism

As previously discussed by [27], at this region due to the small magnitude of the Weber number, defined as $We=\rho v^{2}d/\sigma$, the kinetic force can be ignored. The force balance is written as in Equation (7).

(7) $$F_{\sigma_{j}}=F_{g}$$

where the gravity and surface tension forces can be rewritten as Equation (8) and Equation (9), respectively. The subscript “*j* ” can take on the values of either *i* or *m*, referring to the fact that $\sigma_{i}$ here refers to the interfacial tension between the middle and inner phases ($\sigma_{i}=\sigma_{im}$).

(8) $$F_{g}=\rho Q_{j}gt$$

(9) $$F_{\sigma_{j}}=2\pi r\sigma_{j}cos\theta_{j}\left(t\right)$$

## 5. Results

### 5.1. Final Morphology of Droplets

Following the separation of the inner and middle phases at the capillary tip, the resultant emulsion morphologies within the container exhibit variations that are contingent upon the interfacial tension ratios of the respective phases within the container. $\sigma_{io}$ is the interfacial tension between inner and outer phase, $\sigma_{im}$ is the interfacial tension between inner and middle phases, and $\sigma_{mo}$ is the interfacial tension between middle and outer phases. The supplied figures in Table 1 illustrate the equilibrium topologies of droplets in our experiments, which are determined based on the ratios of interfacial tension. Below is a detailed description of the two total and partial engulfment instances that were observed.

#### 5.1.1. Complete Engulfment

**Figure 5:**
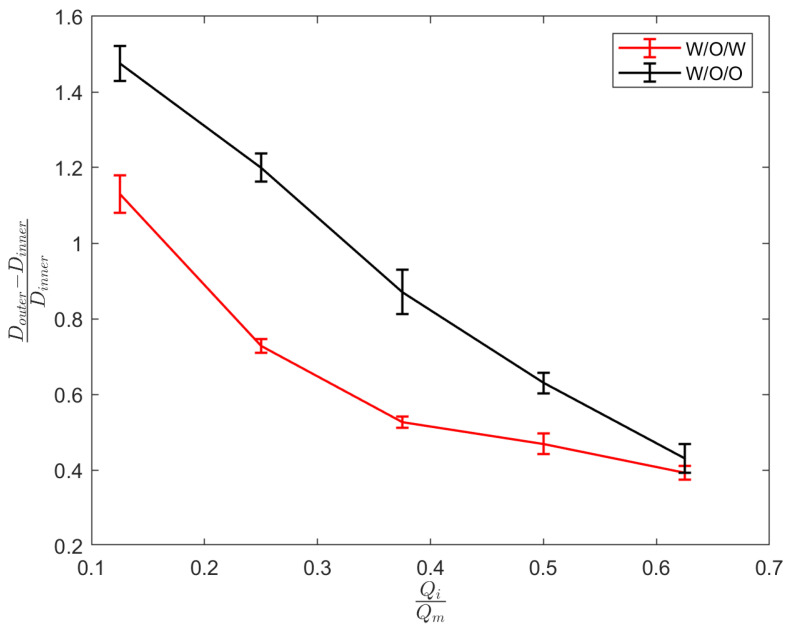
Shell thickness of core–shell droplets variation with flow rate ratio. Data points represent the mean value of 8 experimental results and error bars show the variability in the data.

**Figure 6:**
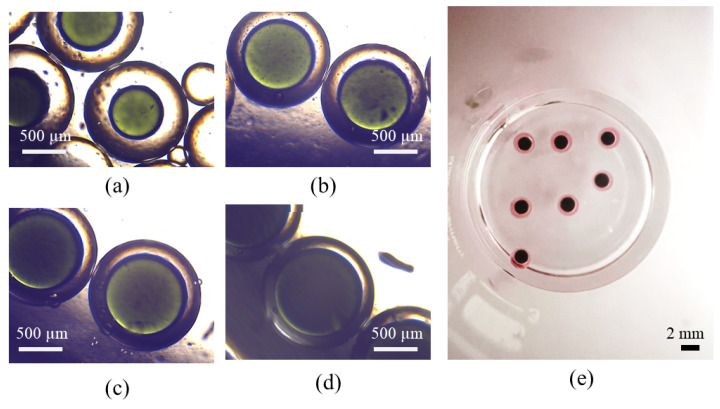
Final morpholgy of (**a**–**d**) w/o/w and (**e**) w/o/o emulsions. $Q_{m}$ = 80 μL/min is fixed through the whole experiment while the core size is controlled by $Q_{i}$.

Double droplets, showcasing the full encapsulation of the inner phase within the middle phase, are produced using either water-in-oil-in-oil (w/o/o) or water-in-oil-in-water (w/o/w) emulsions. In the case of generating (w/o/o) emulsions, the inner phase comprises DI water, the middle phase is composed of sesame oil, and the outer phase is constituted by silicone oil. For the formation of (w/o/w) emulsions, the inner phase consists of DI water, the middle phase involves HFE oil, and the outer phase is a solution of 1% PVA in water. PVA is incorporated into the external phase to ensure the emulsion’s long-term stability. Throughout our experimental trials, the flow rate of the middle phase is consistently maintained at a fixed value of $Q_{m}$ = 80 μL/min. Adjusting the core size is accomplished by varying the flow rate of the inner phase. The adjustment of the core size is achieved through the manipulation of the flow rate of the inner phase. The regulation of the thickness of the shell is determined by the ratio of flow rates, as seen in Figure 5. The minimum dimensionless shell thickness is observed when the ratio of flow rates between the inner and middle regions is maximized. Shell thickness control of single-core w/o/w emulsion is demonstrated in Figure 6 when the inner phase flow rate is increased from (a) $Q_{i}$ = 10 μL/min to (b) $Q_{m}$ = 20 μL/min, (c) $Q_{m}$ = 40 μL/min, and $Q_{m}$ = 50 μL/min. Figure 6e shows the final equilibrium state of w/o/o emulsion made by $Q_{i}$ = 10 μL/min, and $Q_{m}$ = 80 μL/min.

#### 5.1.2. Partial Engulfment

During the fabrication process of an oil-in-water-in-oil (o/w/o) emulsion, the inner phase consists of sesame oil, the middle phase is composed of an aqueous solution, and the outer phase is silicone oil. The phenomenon of partial engulfment is observed during this process, which can be attributed to the interfacial tension ratios that satisfy a Neumann triangle. Consequently, Janus structures can be created using the suggested device in a single step.

### 5.2. Droplet Size Prediction

The videos captured during the experimental testing were subjected to manual analysis to determine the frequency of droplet production. By utilizing the frequency, $f_{m}$, (or period $\tau_{m}$) at which the middle phase droplet production occurs, it becomes possible to compute the volume of the droplet, whether it is the inner or middle droplet, using Equation (10) where the variable “*j* ” can represent either “*i* ” or “*m* ”. The period of the middle phase droplet formation is mainly influenced by the value of $Q_{m}$, with the exception of cases where the inner phase is water and exhibits favorable wettability with the capillary surface. In such instances, the duration of middle-phase droplet formation is affected by both $Q_{m}$ and $Q_{i}$.

It should be noticed that the requirement of double droplet detachment is $\tau_{m}<\tau_{i}$ ($\tau_{i}$ is the inner phase period). In this case, having $V_{t}=V_{i}+V_{m}$ as the total volume of the droplet, the droplet radius is calculated as $R_{t}={\left(3V_{t}/4\pi \right)}^{1/3}$. The thickness of the shell in the core–shell structure can be determined using Equation (11) when the detached double droplet is fully engulfed by the outer phase of the container.

(10) $$V_{j}=\frac{Q_{j}}{f_{m}}=Q_{j}\tau_{m}$$

(11) $$t_{shell}=R_{t}-R_{i}=0.62\tau_{m}^{1/3}\left({\left(Q_{m}+Q_{i}\right)}^{1/3}-{\left(Q_{i}\right)}^{1/3}\right)$$

Equations (10) and (11) apply to both Case 1 and Case 2. If $\tau_{m}>\tau_{i}$, in Case 1, in which the formation of the double droplet is at region 2, the observed regime is either the decussate regime or multiple core regimes. In the decussate regime, single and double droplets appear regularly in this regime [28]. Tailored emulsion with multiple cores, will be obtained with n = $\left\lceil \tau_{m}/\tau_{i}\right\rceil$ single droplets of the inner phase, n−1 core droplets having the volume of $Q_{i}$$\tau_{i}$, and the volume of the last core will be equal to $Q_{i}\left(\tau_{m}-\left\lfloor \tau_{m}/\tau_{i}\right\rfloor \tau_{i}\right)$. The volume of the middle phase droplet is $Q_{m}$$\tau_{m}$. In Case 2, only the decussate regime is observed. The number of single droplets of the inner phase is estimated as $n=\lfloor \tau_{m}/\tau_{i}\rfloor$ and the shell thickness of the produced double droplet, in this case, is calculated by Equation (12).

(12) $$t_{shell}=0.62\tau_{m}^{1/3}{\left(\left(Q_{m}+Q_{i}-\left\lfloor \frac{\tau_{m}}{\tau_{i}}\right\rfloor Q_{i}\frac{\tau_{i}}{\tau_{m}}\right)\right)}^{1/3}-{\left(Q_{i}-\left\lfloor \frac{\tau_{m}}{\tau_{i}}\right\rfloor Q_{i}\frac{\tau_{i}}{\tau_{m}}\right)}^{1/3})$$

**Figure 7:**
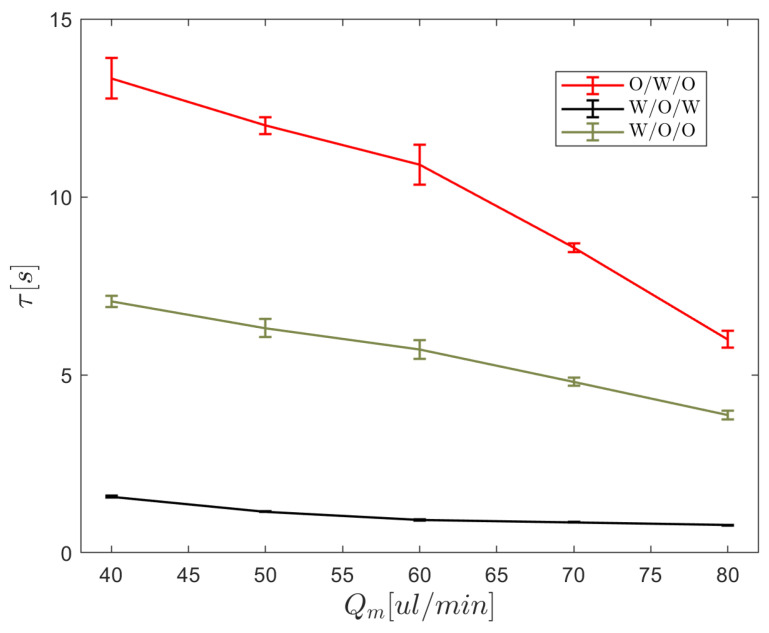
The duration of the middle phase droplet formation period exhibits variability in response to changes in the flow rate of the middle phase. The data points represent the average obtained from three separate experimental tests and included error bars visually convey the uncertainties associated with these values.

**Figure 8:**
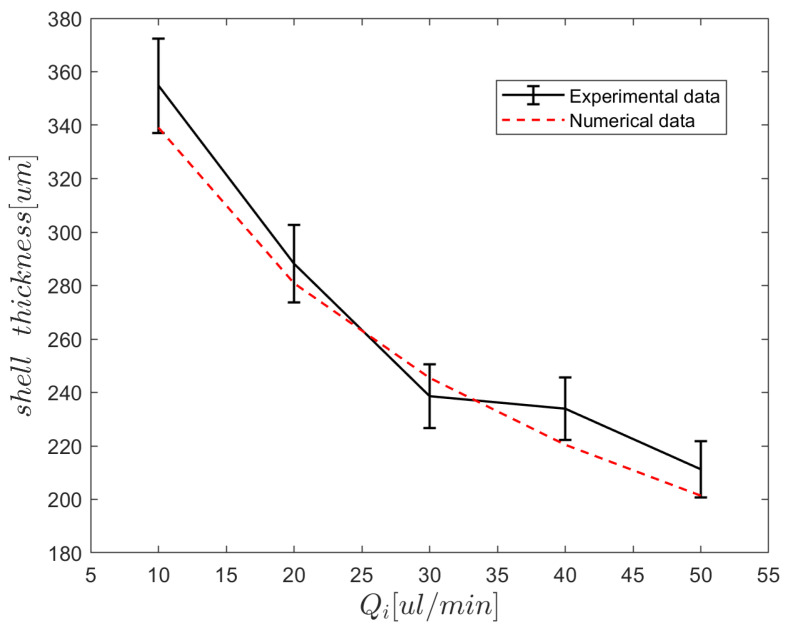
The comparison between the numerical estimation of shell thickness and the experimental results in w/o/w formation. The data points signify the mean values derived from five distinct experimental trials.

Figure 7 depicts the changes in the period of middle phase droplet production with middle phase flow rate without taking the inner phase into account. This pattern will undoubtedly change if the inner phase is also included or the geometry of the capillary tip is changed. If the middle phase is a low-viscosity oil, such as HFE-7500, the duration of droplet production is short, the frequency is high, and droplet volumes are smaller. The frequency will decrease if high-viscosity oil is used as the middle phase. The frequency is at its lowest magnitude when water is used as the middle phase, which has favorable wetting with the capillary surface. We adjusted the flow rate of the middle phase to $Q_{m}=80\;\mu L/\min$, with a corresponding value of $\tau_{m}=0.779$, and conducted a repeated experiment to generate droplets. We measured the diameters of the resulting droplets to calculate the thickness of the shell in the w/o/w formation. Simultaneously, we calculated the predicted shell thickness in the w/o/w formation using Equation (11). The comparison between the experimental and predicted results demonstrated good agreement, with an average error of less than 5%, as illustrated in Figure 8.

### 5.3. On-Demand Complex Emulsions

**Figure 9:**
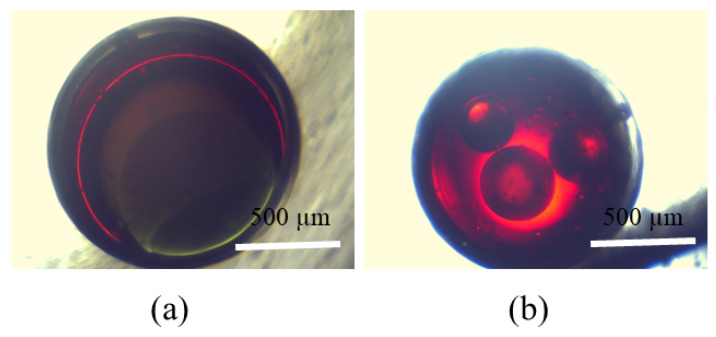
(**a**) Three layered droplets; (**b**) Three core droplets simply fabricated using the apparatus.

To further substantiate the efficacy of our uncomplicated apparatus, we employed it to generate customized and complex emulsions. By introducing the dental needle into the primary capillary, the equipment can be utilized for the generation of triple emulsions. In this study, we utilized an aqueous inner phase containing green dye, a first middle phase composed of sesame oil with red dye, a second oil phase consisting of HFE oil, and an outside phase comprising a 1% PVA solution in water. These components were combined to generate a triple emulsion, as depicted in Figure 9a. The flow rates are designated as $Q_{i}$ = 30 μL/min, $Q_{m1}$ = 30 μL/min, $Q_{m2}$ = 30 μL/min, and $Q_{m2}$ = 80 μL/min. As documented by [29], tailored emulsions commonly contain from one to seven inner droplets. Our device demonstrates the ability to create this specific emulsion. The tailored emulsion containing multiple cores is fabricated by tuning $\tau_{m}$ and $\tau_{i}$, as illustrated in Figure 9b, with a specified number of three small inner water droplets encapsulated within the substantial oil (HFE-7500) droplet. $\tau_{m}$ can be controlled with flow rate ratio and phase properties. In this case, the inner flow rate is set to $Q_{i}$ = 80 μL/min and the middle phase flow rate is $Q_{m}$ = 40 μL/min. The flow rate of the middle phase is decreased to extend the duration of droplet formation, whilst the flow rate of the inner phase is augmented to shorten the duration of droplet formation. The outer phase in the container is an aqueous solution of 1 wt% PVA.

### 5.4. Hollow Alginate Microparticle

**Figure 10:**
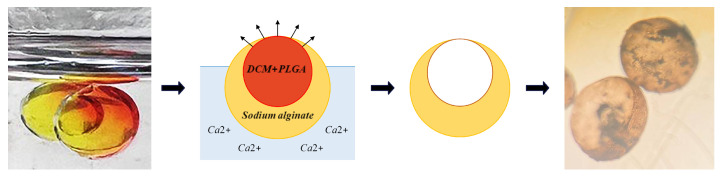
The process of ionic gelation of w/o/o emulsion to fabricate hollow alginate microparticle.

The collection of double droplets within a container affords us the ability to manipulate the gelation process in ion-induced microparticle gelation, hence enhancing our control over this mechanism. When o/w/o or PLGA in DCM/an aqueous solution of alginate/HFE-7500 is created, the aqueous phase travels downwards due to the difference in density between the phases. Meanwhile, PLGA dissolved in DCM comes into contact with air, and the diffusion of DCM is accelerated. At this time, acetic acid is injected into HFE-7500, which triggers the release of calcium ions and initiates the gelation process of alginate. The concurrent gelation of alginate and solidification of PLGA will yield a non-spherical hollow structure. The resulting hollow microparticle is shown in Figure 10. Numerous studies have explored the efficacy of hollow microparticles in improving the release of encapsulated compounds when compared to solid microparticles [23]. Our proposed approach provides a straightforward alternative for the fabrication of these complex structures.

### 5.5. Conclusions

We have demonstrated a single capillary-based on-demand droplet generating device for precisely controlled emulsion generation. The process of on-demand droplet production relies on the interplay between gravity, surface tension, and kinetic forces. Upon analyzing the force equilibrium during the droplet pinch-off phenomenon, it has been determined that the kinetic force exhibits a relatively lesser size when compared to other forces involved, hence rendering it negligible. The significance of core–shell droplets, together with their numerous uses, prompted us to investigate the impact of several parameters such as droplet formation frequency and the flow rate ratio between the inner and middle phases on the thickness of the shell. To demonstrate the device’s further capabilities, triple emulsion and tailored emulsion with multiple cores are experimentally created. Furthermore, by using the disparity in density within the container, it is possible to determine the location of the core in core–shell droplets. Subsequently, the process of gelation in the emulsion can result in the creation of designed microparticles, such as hollow alginate microparticles.

## Figures and Tables

**Table 1:** The process of complete or partial engulfment of the inner and middle phases is determined by the interfacial tension ratios between the three phases.

Emulsion	Criteria	Sample
W/O/O Water/Silicon oil/Sesame oil	$\sigma_{mo}/\sigma_{mi}+\sigma_{io}/\sigma_{mi}<1$	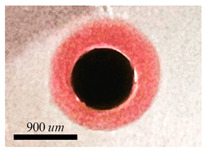
W/O/W Water/HFE/PVA 1 wt%	$\sigma_{mo}/\sigma_{mi}+\sigma_{io}/\sigma_{mi}<1$	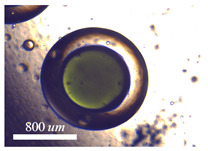
O/W/O Sesame oil/Water/Silicone oil	$|\left(\sigma_{mo}/\sigma_{mi}\right)-\left(\sigma_{io}/\sigma_{mi}\right)|\;<\;1$ & $\sigma_{mo}/\sigma_{mi}+\sigma_{io}/\sigma_{mi}>1$	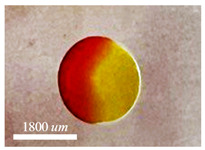

## Data Availability

The data presented in this study are available on request from the corresponding author. The data are not publicly available due to the ongoing nature of our research.
